# Supplementary material for: Asymmetric Synthesis of Three Alkenyl Epoxides: Crafting the Sex Pheromones of the Elm Spanworm and the Painted Apple Moth
Source: Molecules. 2024 May 4;29(9):2136. doi: 10.3390/molecules29092136 (PMC11085616; doi:10.3390/molecules29092136)
Supplement: Supplementary file 1 [file molecules-29-02136-s001.zip › molecules-2985171-supplementary.pdf]

Supporting Information for
Asymmetric Synthesis of Three Alkenyl Epoxides: Crafting the Sex Pheromones of the Elm Spanworm and the Painted Apple Moth

Yun Zhou [1], JiananWang [2], Beijing Tian [1], Yanwei Zhu [1], Yujuan Zhang [1], Jinlong Han [1], Jiangchun Zhong [2] and Chenggang Shan [1,*]

[1] Institute of Industrial Crops, Shandong Academy of Agricultural Sciences, Jinan 250100, China;

zysass2021@163.com (Y.Z.); tianbeijing07@126.com (B.T.); zhuyanwei96@163.com (Y.Z.);

zhangyujuan@caas.cn (Y.Z.); goldendragonh@163.com (J.H.)

[2] Department of Applied Chemistry, China Agricultural University, Beijing 100193, China;

xxjnwang@163.com (J.W.); zhong@cau.edu.cn (J.Z.)

* Correspondence: shanchenggang@126.com; Tel.: +86-053166655005

Table of Contents

1. General information……………………………………………………………………….......S2

2. Synthesis of Mosher esters of chiral epoxy alcohols……………………..…………………….……………S2

3. ^{1}H, ^{13}C spectra of the products…………………………………………………..S4

4. Reference……………………………………..………..S22

1. General information

All reactions were carried out within a Schlenk line system under an inert atmosphere of argon. Commercially available reagents were utilized as received, without additional purification. In contrast, solvents underwent distillation following standard procedures prior to use. Column chromatography was generally performed on a silica gel (200–300 mesh) and eluting with petroleum ether and ethyl acetate. ^{1}H and ^{13}C NMR spectra were recorded on a Bruker DP-X500 MHz spectrometer. Chemical shifts were reported in ppm relative to internal tetramethylsilane for ^{1}H NMR and $CDCl_3$ (77.16 ppm) for ^{13}C NMR. High-resolution mass spectra (HRMS) were collected on Waters LCT Premier™ with an ESI mass spectrometer. Optical rotations were determined by a Rudolph Research Analytical AUTOPOL- IV. Melting points were measured on a STUART-SMP3 Melt-Temp apparatus without correction.

2. Synthesis of Mosher esters of chiral epoxy alcohols

Scheme S1. Synthesis of Mosher esters **21-23**.

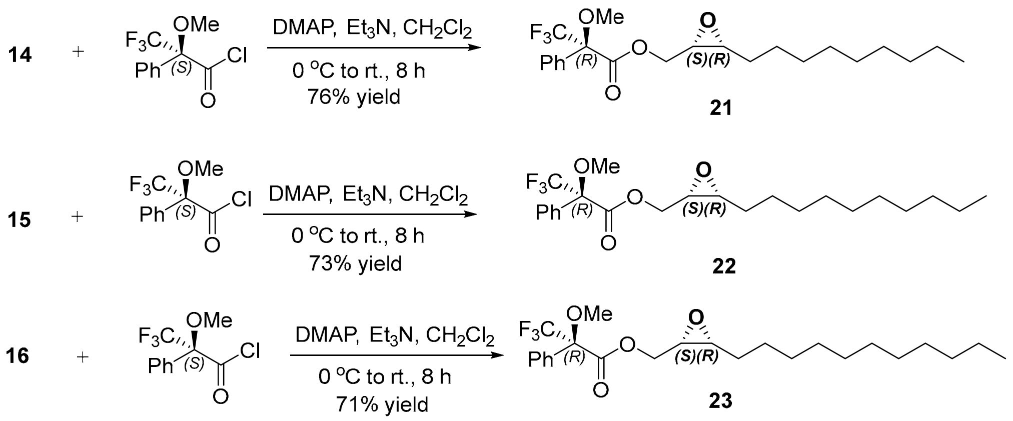

*((2S,3R)-3-nonyloxiran-2-yl) methyl (R)-3,3,3-trifluoro-2-methoxy-2-phenylpropanoate (**21**)*

Alcohol **14** (10.0 mg, 0.05 mmol, 1.0 equiv) was added slowly to the solution of DMAP (6.1 mg, 0.05 mmol, 1.0 equiv) and triethylamine (25.3 mg, 0.25 mmol, 5.0 equiv) in CH_2Cl_2 (2 mL) at 0 °C. (*S*)-(-)-α-Methoxy-α-(trifluoromethy1) phenylacetyl chloride (MTPACl) (25.3 mg, 0.1 mmol, 2.0 equiv) was then added, and the reaction mixture turned yellow. The reaction mixture was allowed to warm to 25 °C and stirred continuously for 8 hours. Subsequently, the reaction was quenched by the addition of 3 mL of water, followed by the separation of the organic phase. The aqueous phase was extracted with CH_2Cl_2 (3× 10 mL). The combined organic layers were washed with brine (10 mL), dried over anhydrous Na_2SO_4, and concentrated under reduced pressure to give crude product. The crude product was purified by thin layer chromatography

to afford ((2*S*,3*R*)-3-nonyloxiran-2-yl) methyl (*R*)-3,3,3-trifluoro-2-methoxy-2-phenylpropanoate (**21)** (15.8 mg, 76% yield) as yellow oil [1, 2]. $[\alpha]_D^{20}$+32.64 (*c* 1.67, $CHCl_3$). ^{1}H NMR (500 MHz, $CDCl_3$) δ 7.54-7.53 (m, 2H), 7.42-7.41 (m, 3H), 4.45 (dd, *J* = 12.0, 4.5 Hz, 1H), 4.39 (dd, *J* = 12.0, 7.0 Hz, 1H), 3.58 (s, 3H), 3.22-3.19 (m, 1H), 3.04-3.01 (m, 1H), 1.57-1.39 (m, 4H), 1.34-1.27 (m, 12H), 0.88 (t, *J* = 7.0 Hz, 3H). HRMS (ESI) *m*/*z* 439.2081 [M+Na]$^+$ (calcd for $C_{22}H_{31}O_4F_3Na$ 439.2067).

*((2S,3R)-3-decyloxiran-2-yl) methyl (R)-3,3,3-trifluoro-2-methoxy-2-phenylpropanoat (**22**)*

According to the similar procedure for Mosher ester **21**, using alcohol **15** (10.7 mg, 0.05 mmol, 1.0 equiv) and (*S*)-(-)-α-methoxy-α-(trifluoromethy1) phenylacetyl chloride (25.3 mg, 0.1 mmol, 2.0 equiv) afforded product **22** (15.7 mg, 73% yield) as a yellow oil. $[\alpha]_D^{20}$ +30.48 (*c* 1.68, $CHCl_3$). ^{1}H NMR (500 MHz, $CDCl_3$) δ 7.54-7.53 (m, 2H), 7.42-7.41 (m, 3H), 4.45 (dd, *J* = 12.0, 4.5 Hz, 1H), 4.38 (dd, *J* = 12.0, 7.0 Hz, 1H), 3.58 (s, 3H), 3.20 (dt, *J* = 7.0, 4.5 Hz, 1H), 3.04-3.01 (m, 1H), 1.58-1.40 (m, 4H), 1.34-1.27 (m, 14H), 0.88 (t, *J* = 7.0 Hz, 3H). HRMS (ESI) *m*/*z* 453.2225 [M+Na]$^+$ (calcd for $C_{23}H_{33}O_4F_3Na$ 453.2223).

((2*S*,3*R*)-3-undecyloxiran-2-yl)methyl(*R*)-3,3,3-trifluoro-2-methoxy-2-phenylpropanoate (**23**)

According to the similar procedure of synthesis of **21**, using alcohol **16** (11.4 mg, 0.05 mmol, 1.0 equiv) and (*S*)-(-)-α-methoxy-α-(trifluoromethy1) phenylacetyl chloride (25.3 mg, 0.1 mmol, 2.0 equiv) afforded product afforded product **23** (15.8 mg, 71% yield) as a yellow oil. $[\alpha]_D^{20}$ +27.44 (*c* 1.09, $CHCl_3$). ^{1}H NMR (500 MHz, $CDCl_3$) δ 7.54-7.53 (m, 2H), 7.42-7.40 (m, 3H), 4.45 (dd, *J* = 12.0, 4.5 Hz, 1H), 4.38 (dd, *J* = 12.0, 7.0 Hz, 1H), 3.58 (s, 3H), 3.20 (dt, *J* = 7.0, 4.5 Hz, 1H), 3.04-3.00 (m, 1H), 1.58-1.41 (m, 4H), 1.34-1.26 (m, 16H), 0.88 (t, *J* = 7.0 Hz, 3H). HRMS (ESI) *m*/*z* 445.2578 [M+H]$^+$ (calcd for $C_{24}H_{36}O_4F_3$ 445.2560).

3. ^{1}H and ^{13}C spectra of the products

Figure S1. ^{1}H NMR Spectrum of compound **8** (500 MHz, $CDCl_3$)

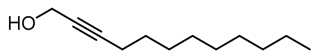

8

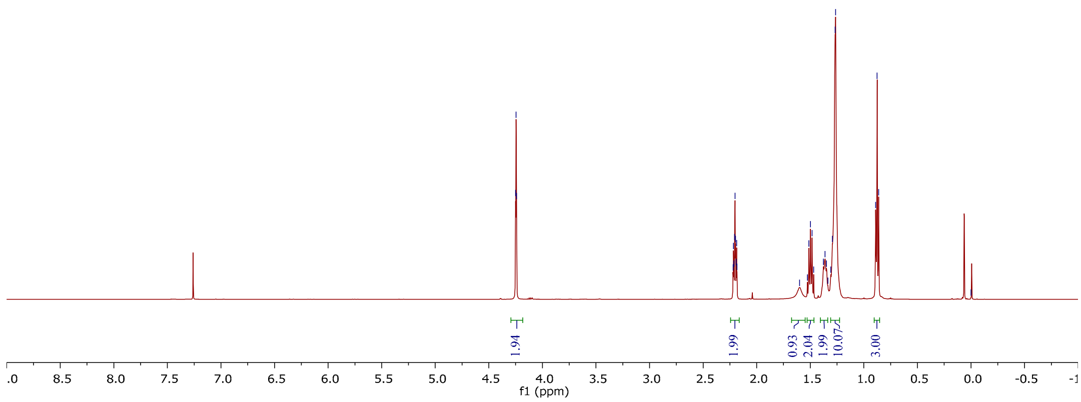

Figure S2. ^{13}C NMR Spectrum of compound **8** (125 MHz, $CDCl_3$)

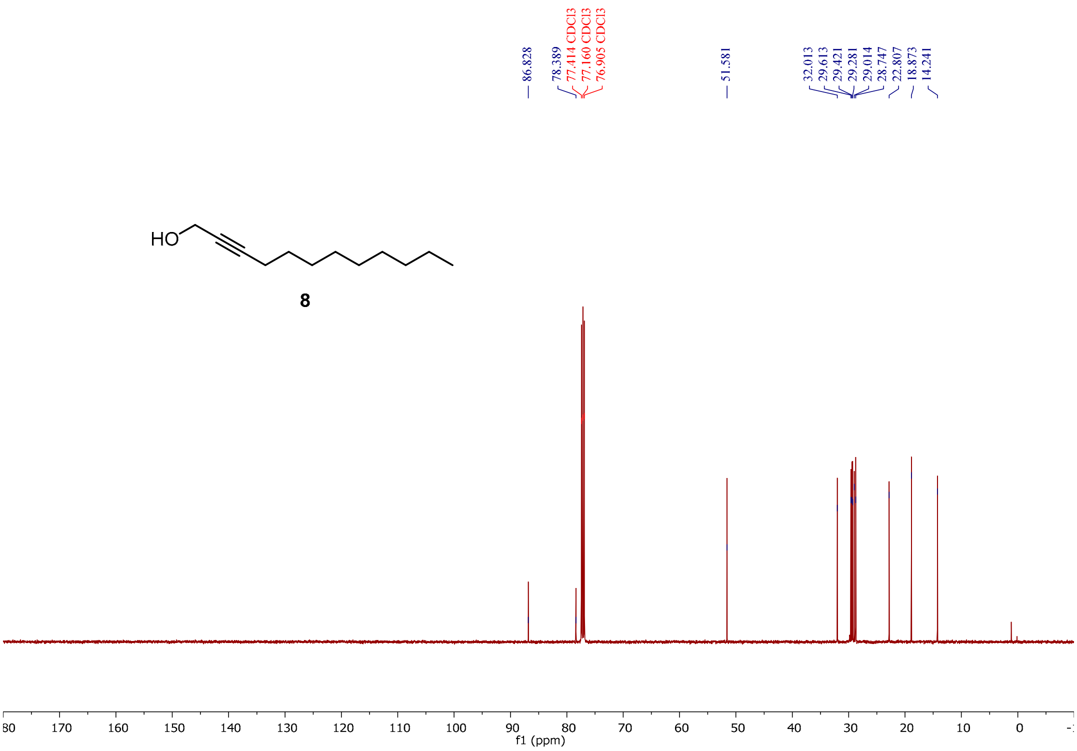

Figure S3. ^{1}H NMR Spectrum of compound **9** (500 MHz, $CDCl_3$)

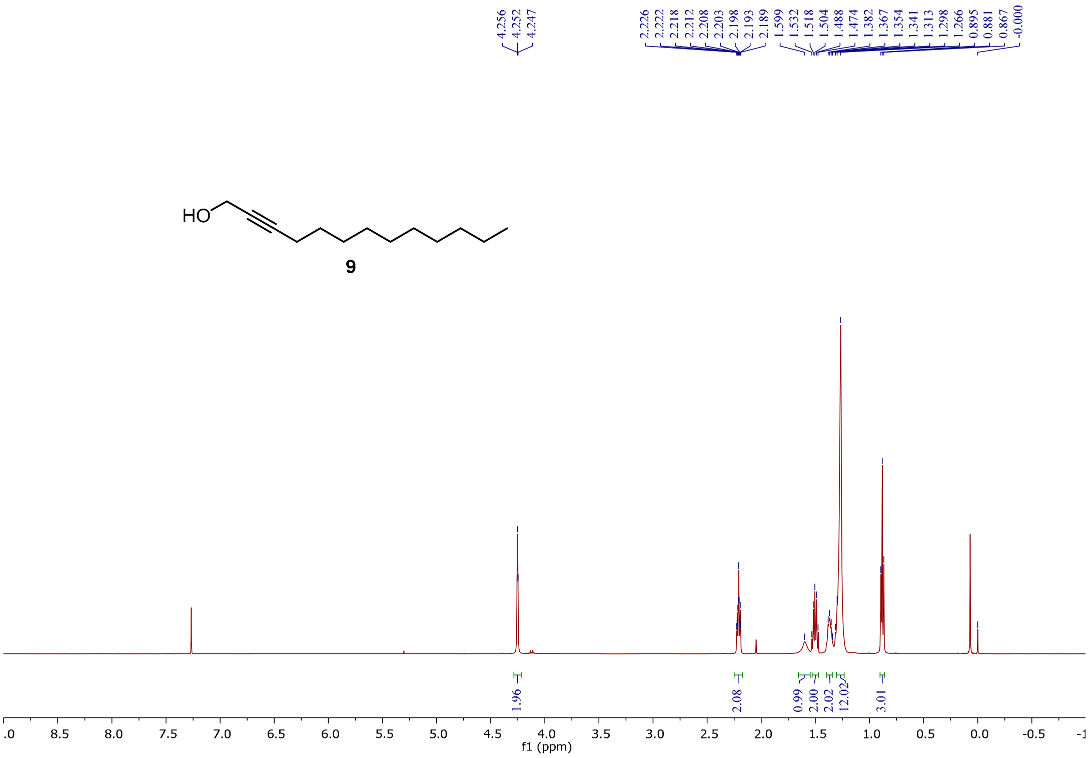

Figure S4. ^{13}C NMR Spectrum of compound **9** (125 MHz, $CDCl_3$)

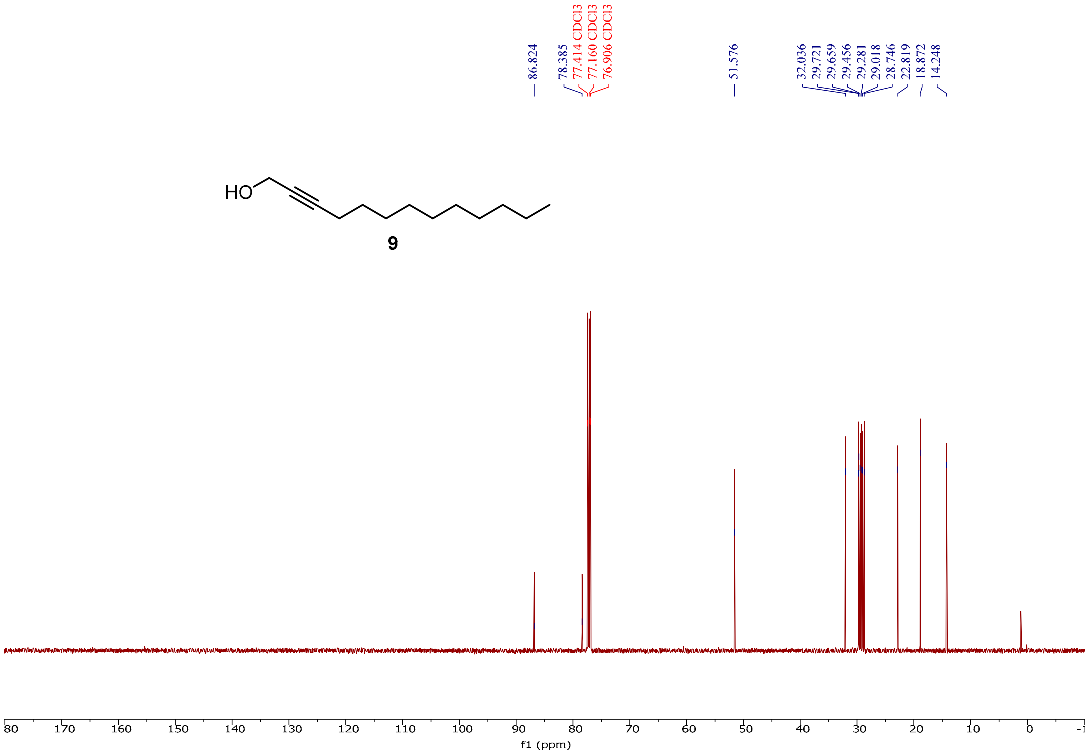

Figure S5. ^{1}H NMR Spectrum of compound **10** (500 MHz, $CDCl_3$)

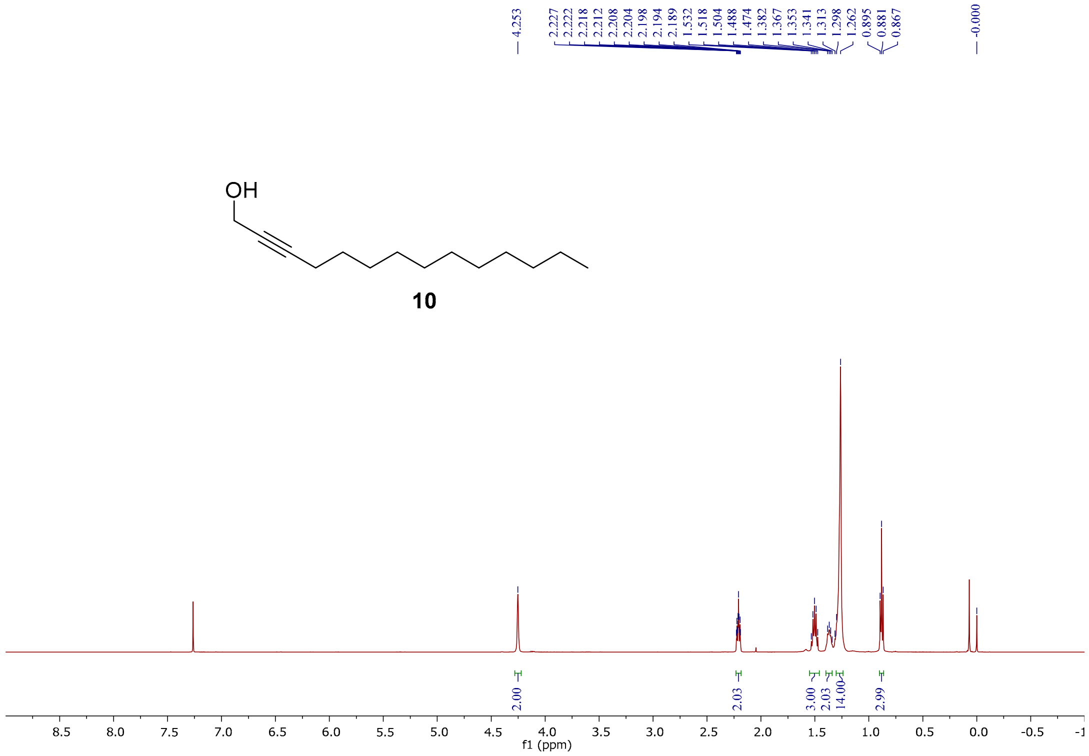

Figure S6 ^{13}C NMR Spectrum of compound **10** (125 MHz, $CDCl_3$)

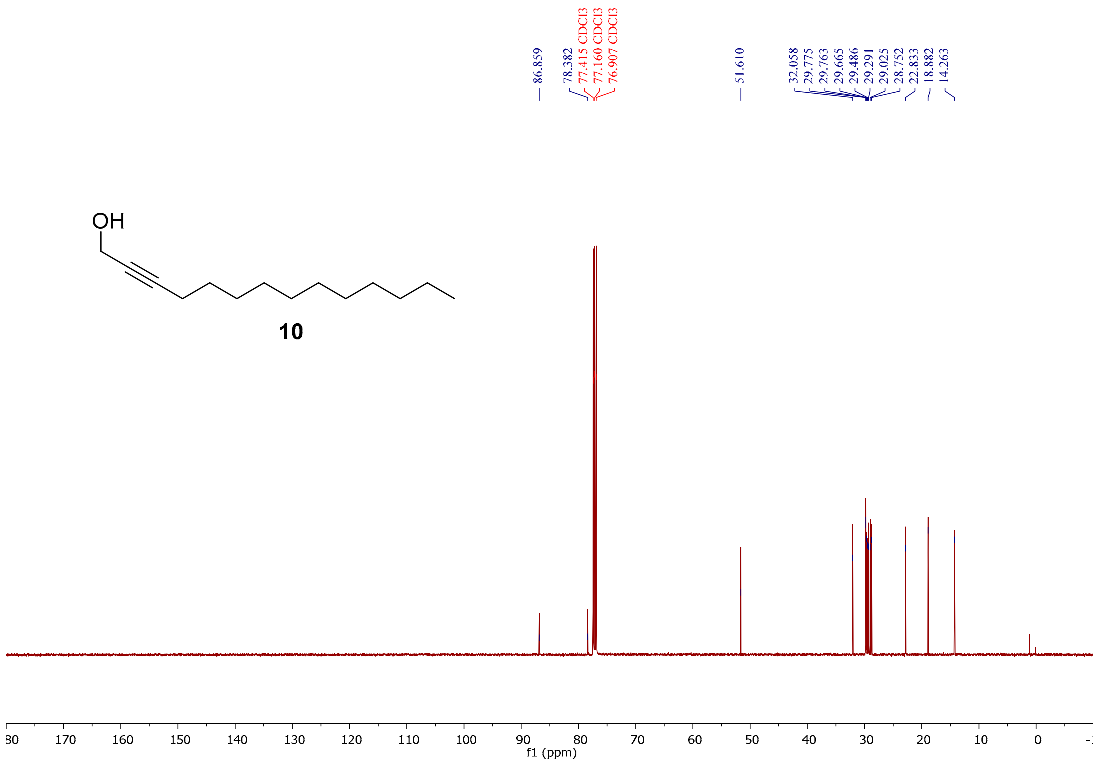

Figure S7. ^{1}H NMR Spectrum of compound **11** (500 MHz, $CDCl_3$)

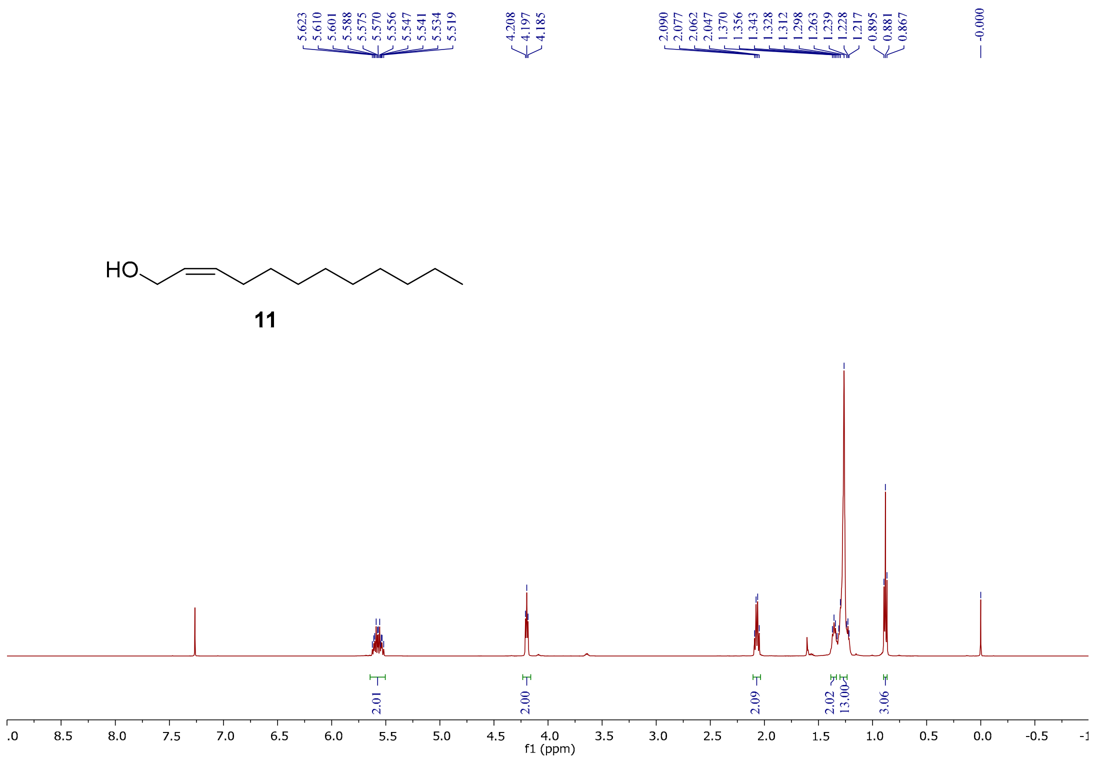

Figure S8 ^{13}C NMR Spectrum of compound **11** (125 MHz, $CDCl_3$)

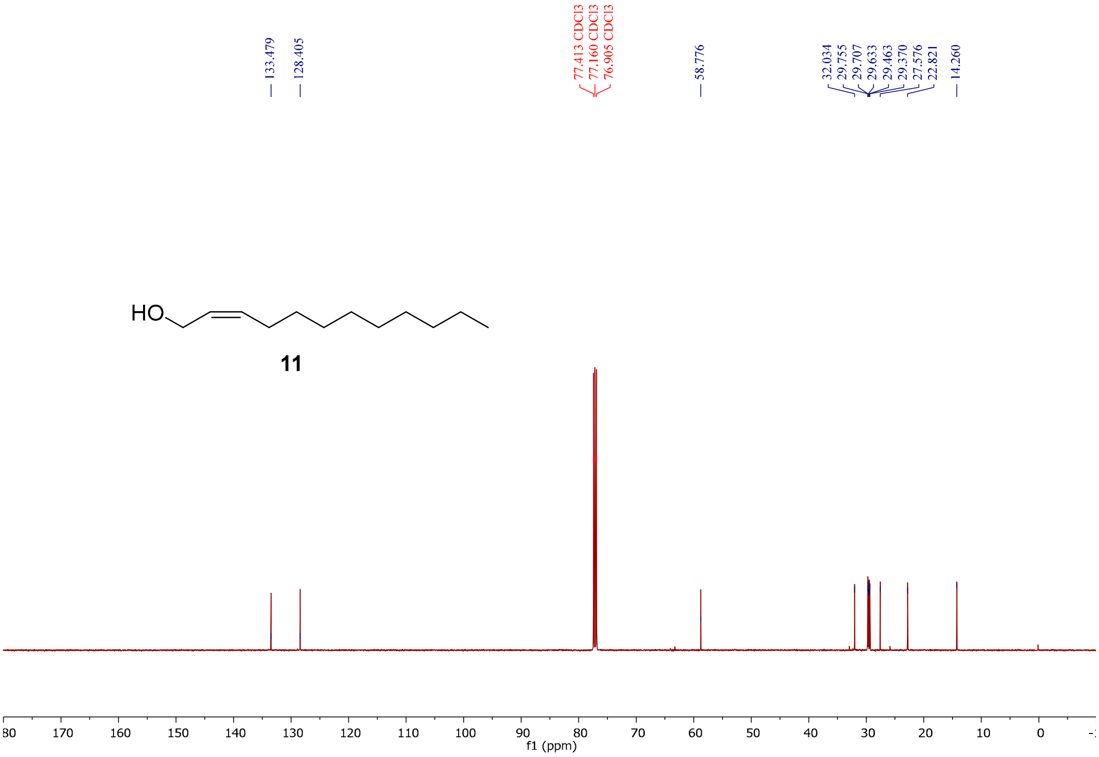

Figure S9. ^{1}H NMR Spectrum of compound **12** (500 MHz, $CDCl_3$)

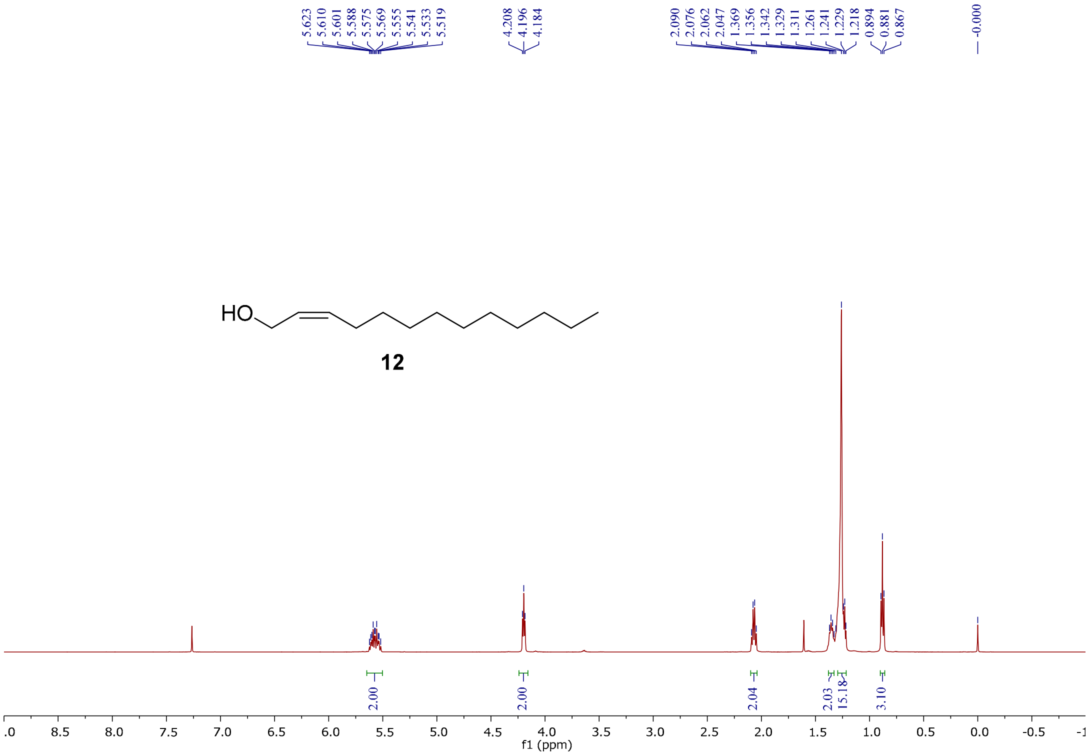

Figure S10 ^{13}C NMR Spectrum of compound **12** (125 MHz, $CDCl_3$)

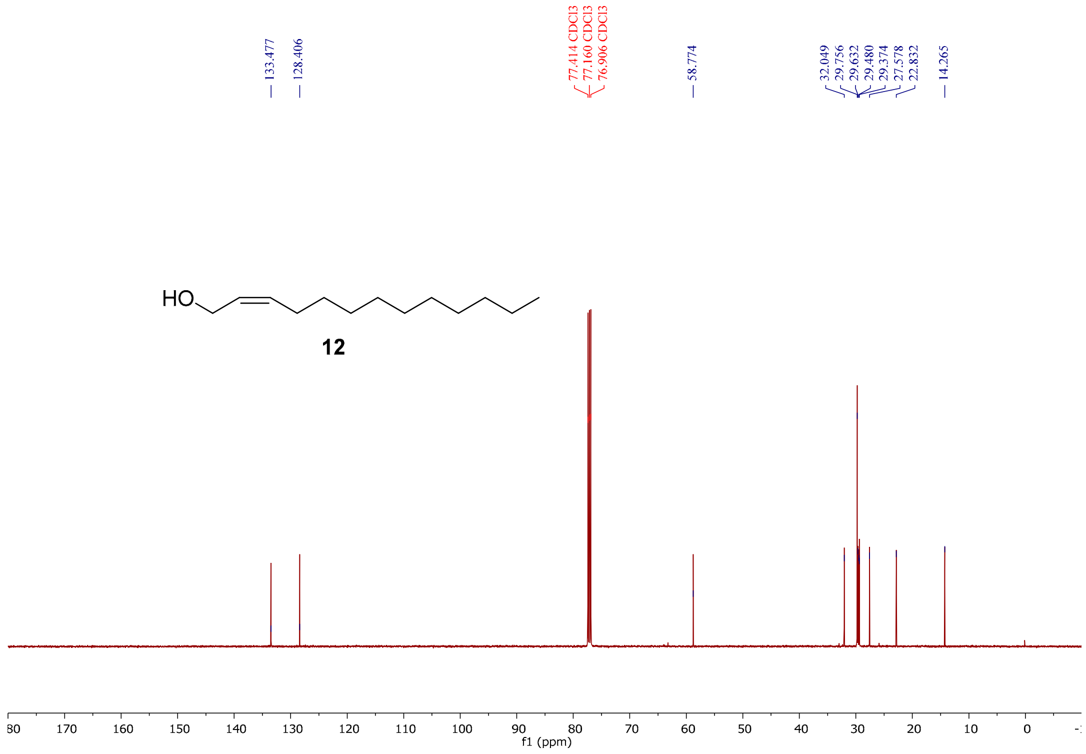

Figure S11. ^{1}H NMR Spectrum of compound **13** (500 MHz, $CDCl_3$)

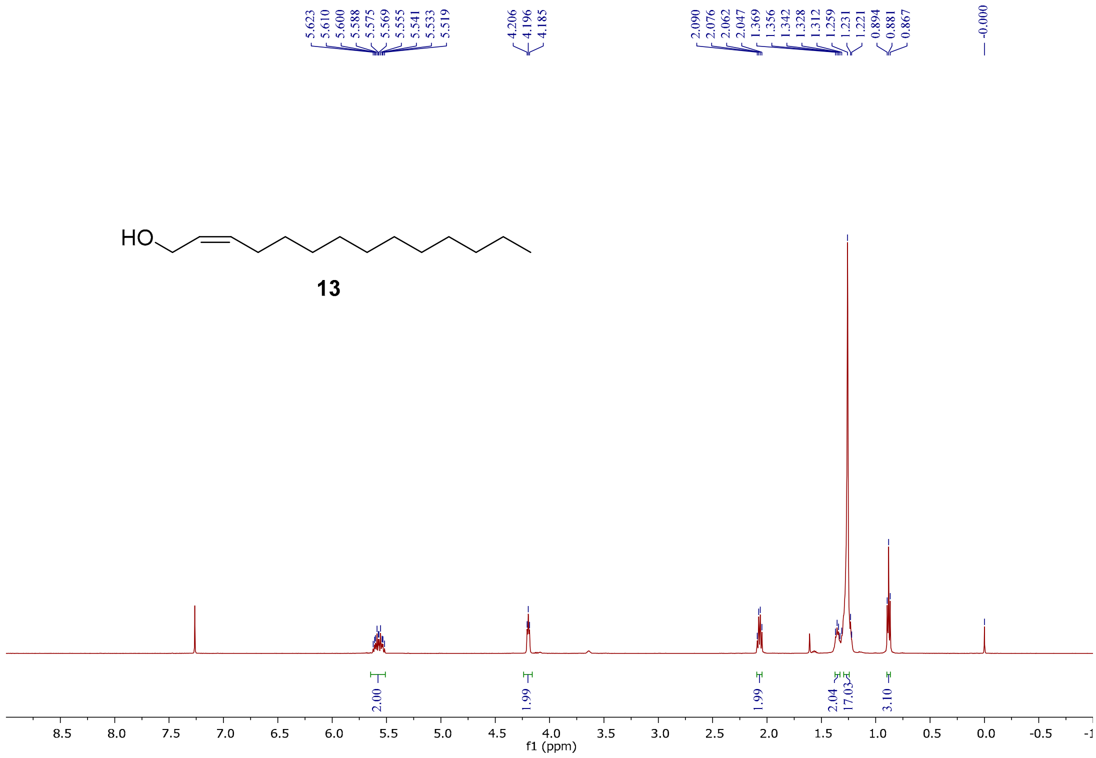

Figure S12 ^{13}C NMR Spectrum of compound **13** (125 MHz, $CDCl_3$)

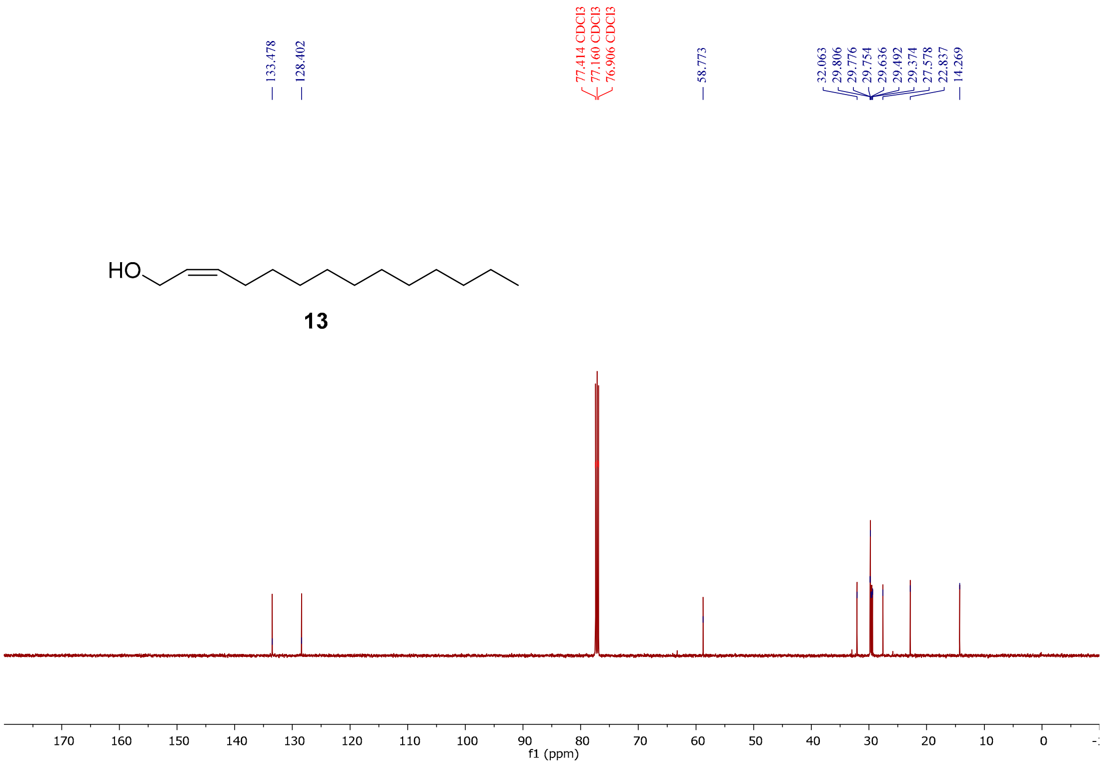

Figure S13. ^{1}H NMR Spectrum of compound **14** (500 MHz, $CDCl_3$)

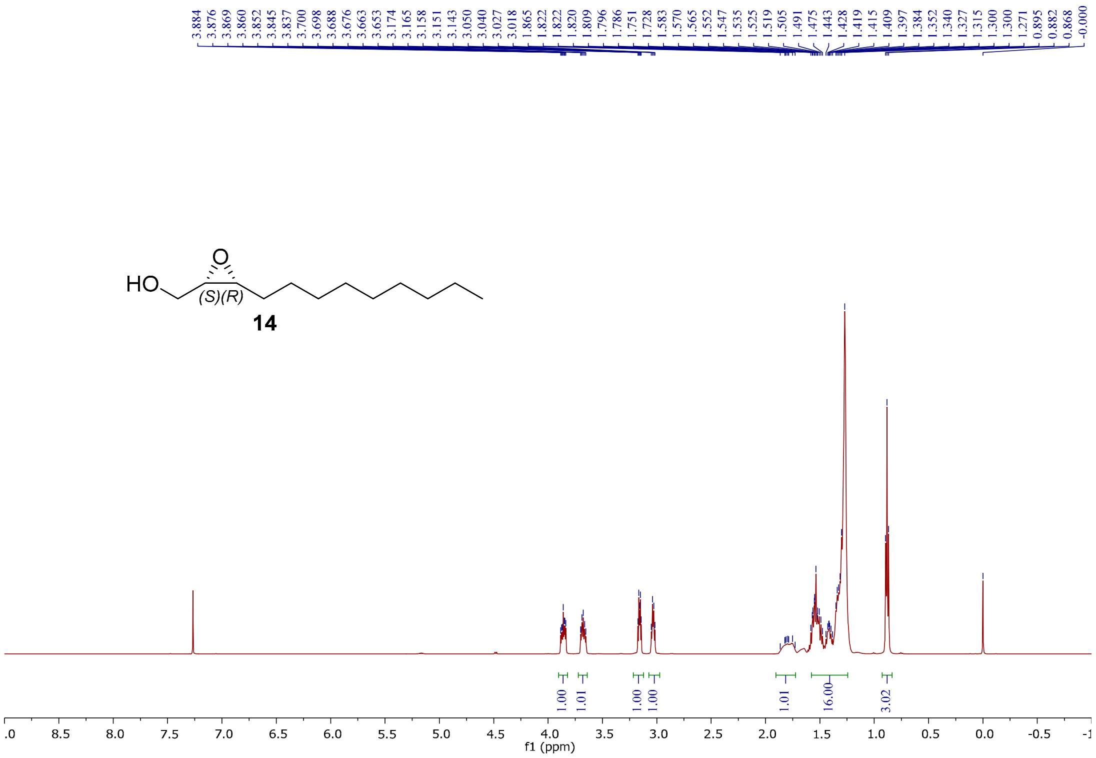

Figure S14 ^{13}C NMR Spectrum of compound **14** (125 MHz, $CDCl_3$)

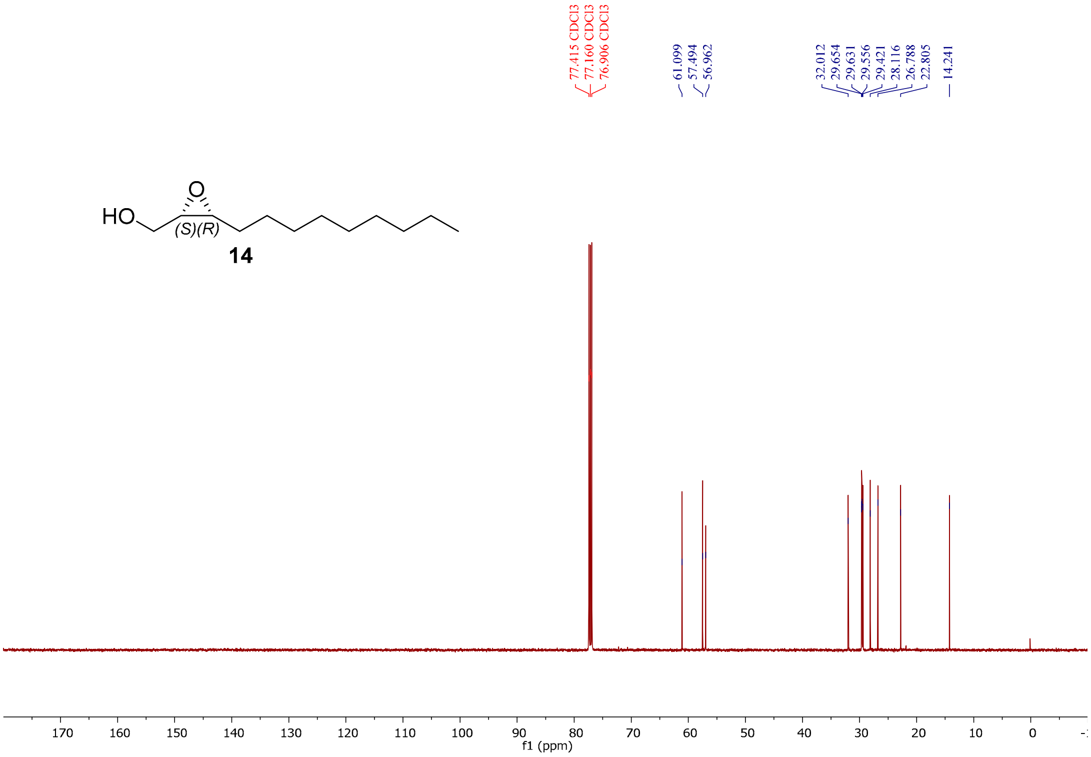

Figure S15. ^{1}H NMR Spectrum of compound **15** (500 MHz, $CDCl_3$)

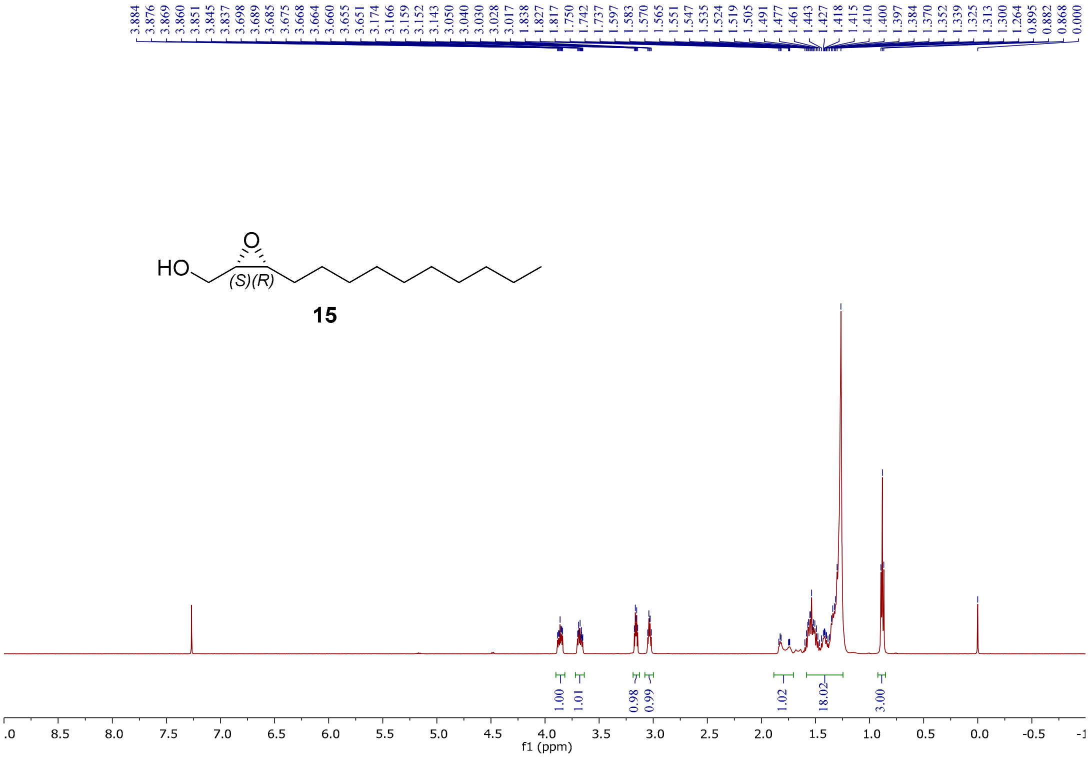

Figure S16 ^{13}C NMR Spectrum of compound **15** (125 MHz, $CDCl_3$)

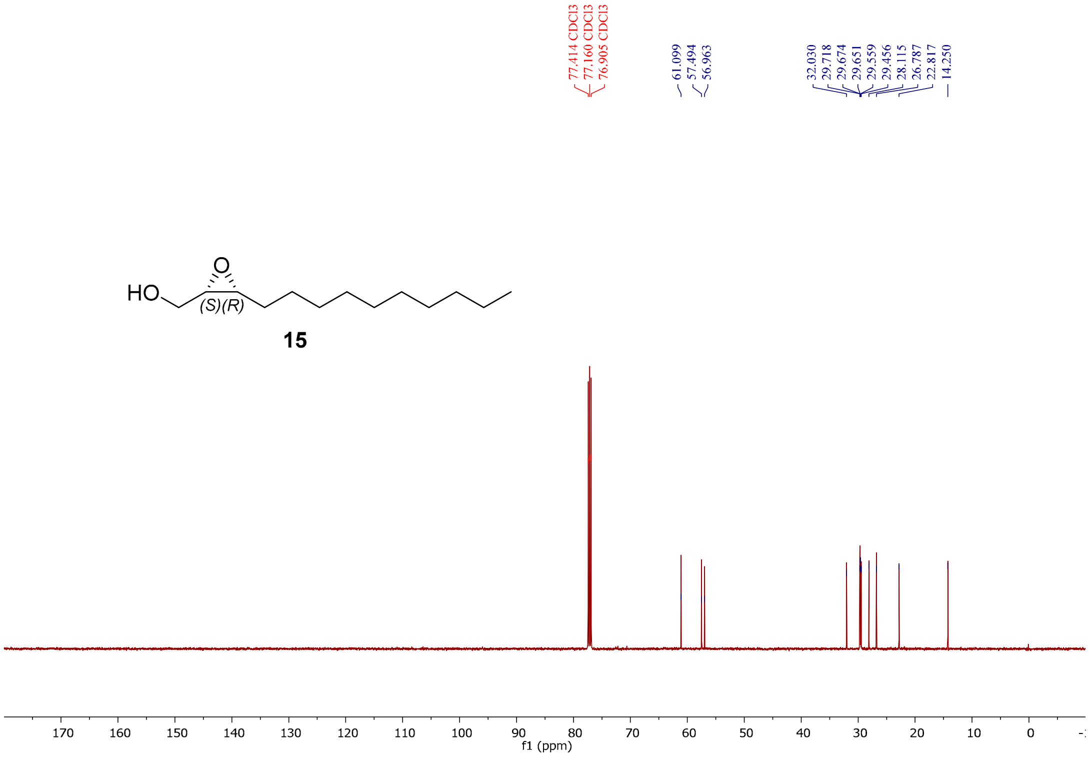

Figure S17. ^{1}H NMR Spectrum of compound **16** (500 MHz, $CDCl_3$)

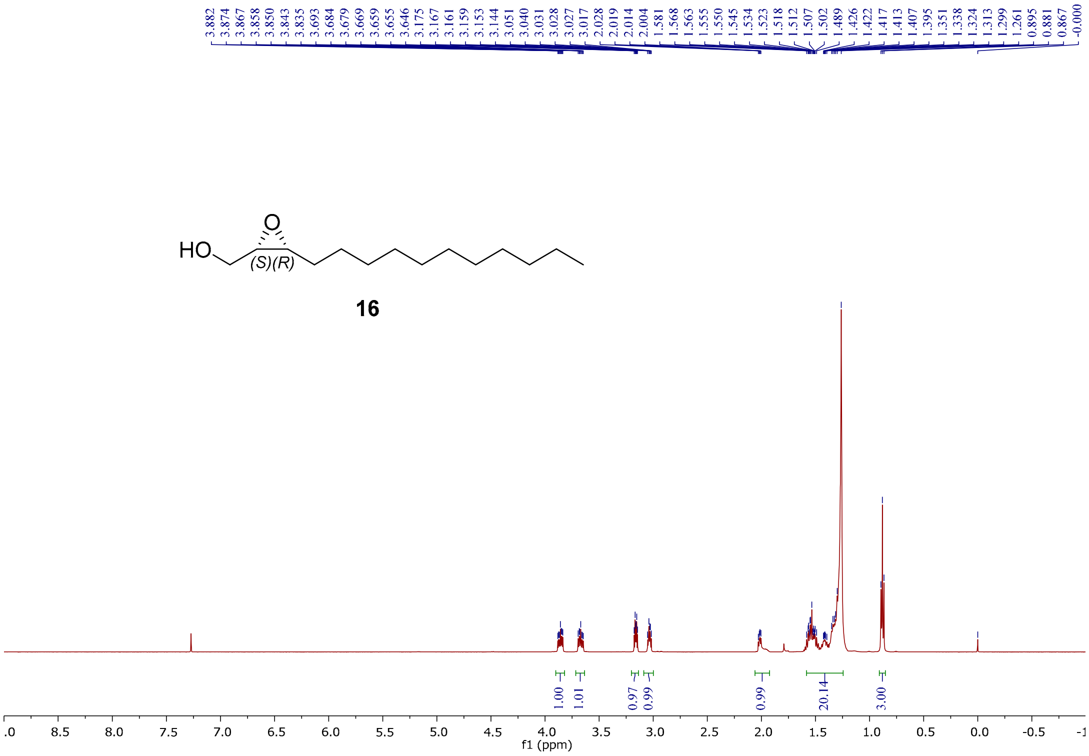

Figure S18 ^{13}C NMR Spectrum of compound **16** (125 MHz, $CDCl_3$)

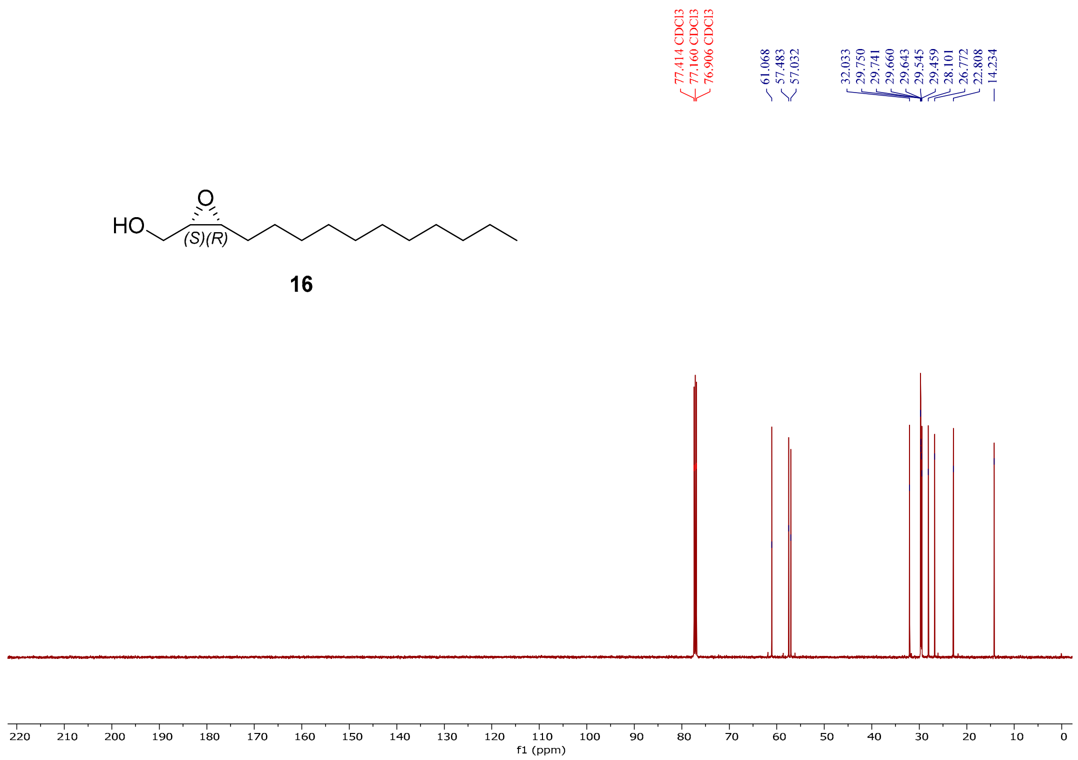

Figure S19. ^{1}H NMR Spectrum of compound **18** (500 MHz, $CDCl_3$)

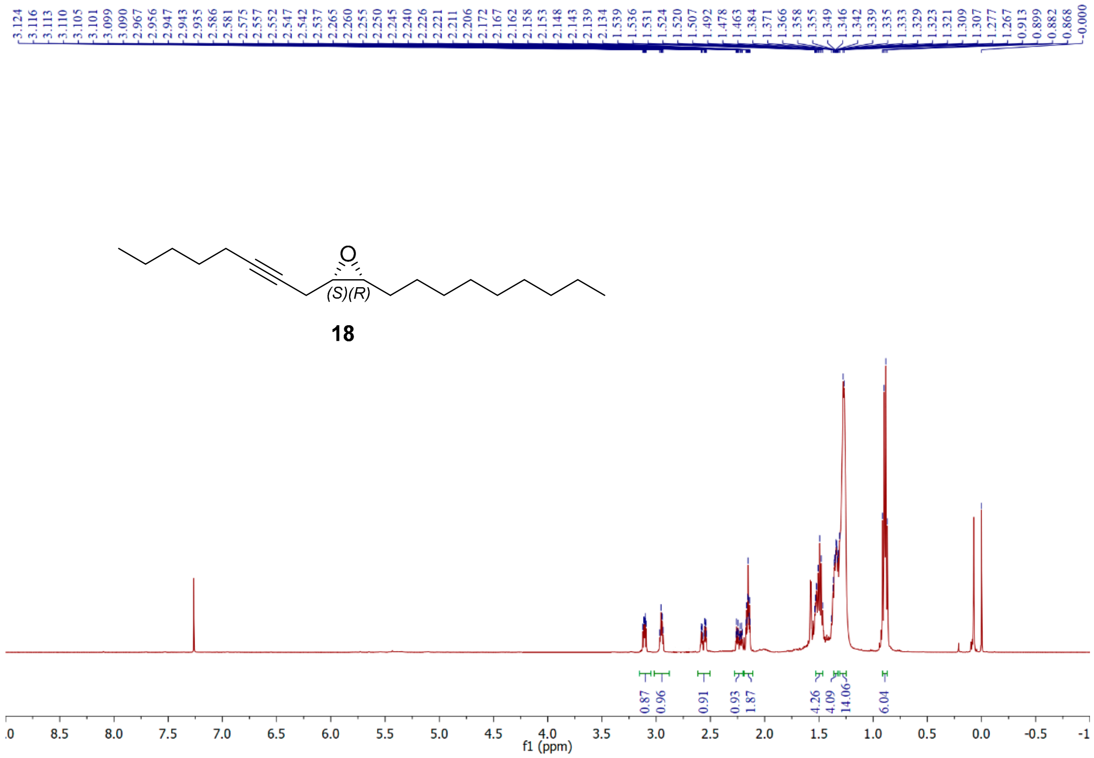

Figure S20 ^{13}C NMR Spectrum of compound **18** (125 MHz, $CDCl_3$)

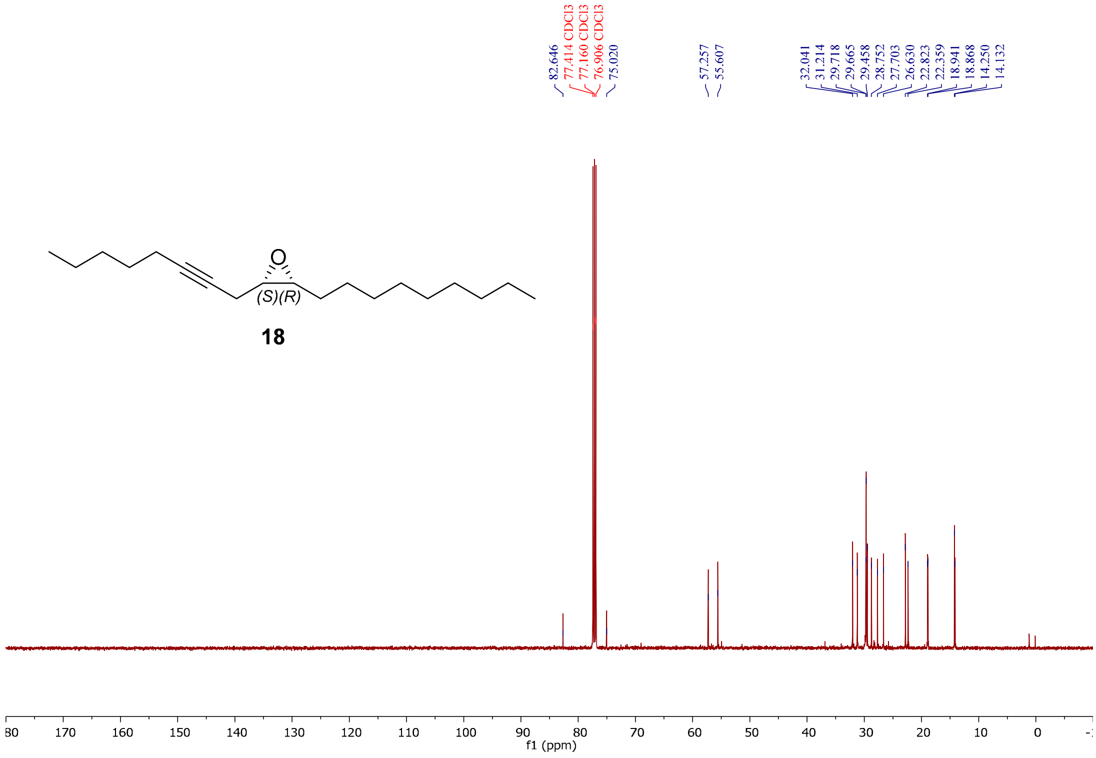

Figure S21. ^{1}H NMR Spectrum of compound **19** (500 MHz, $CDCl_3$)

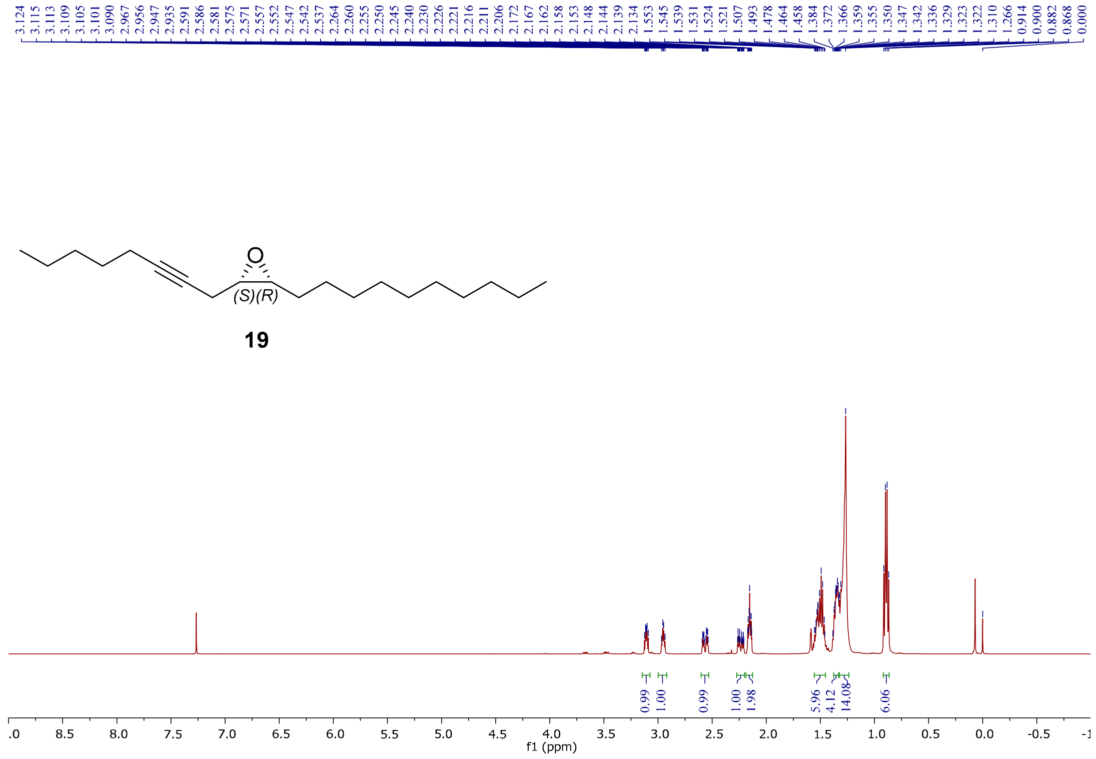

Figure S22 ^{13}C NMR Spectrum of compound **19** (125 MHz, $CDCl_3$)

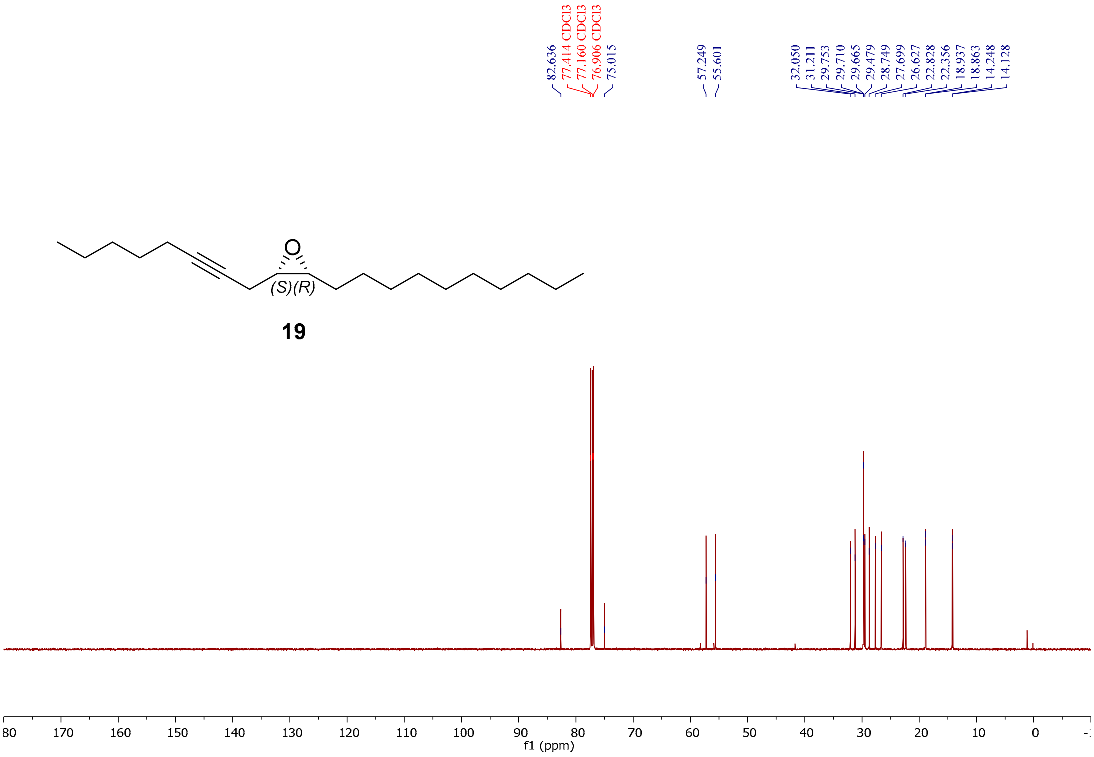

Figure S23. ^{1}H NMR Spectrum of compound **20** (500 MHz, $CDCl_3$)

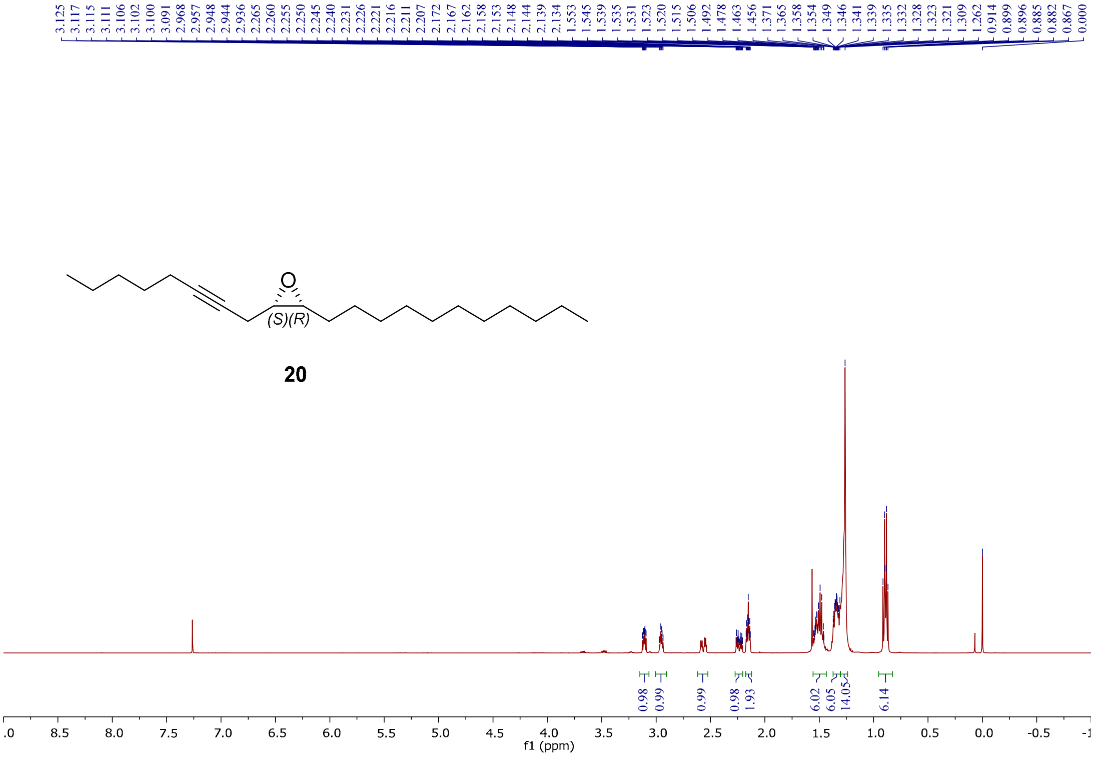

Figure S24 ^{13}C NMR Spectrum of compound **20** (125 MHz, $CDCl_3$)

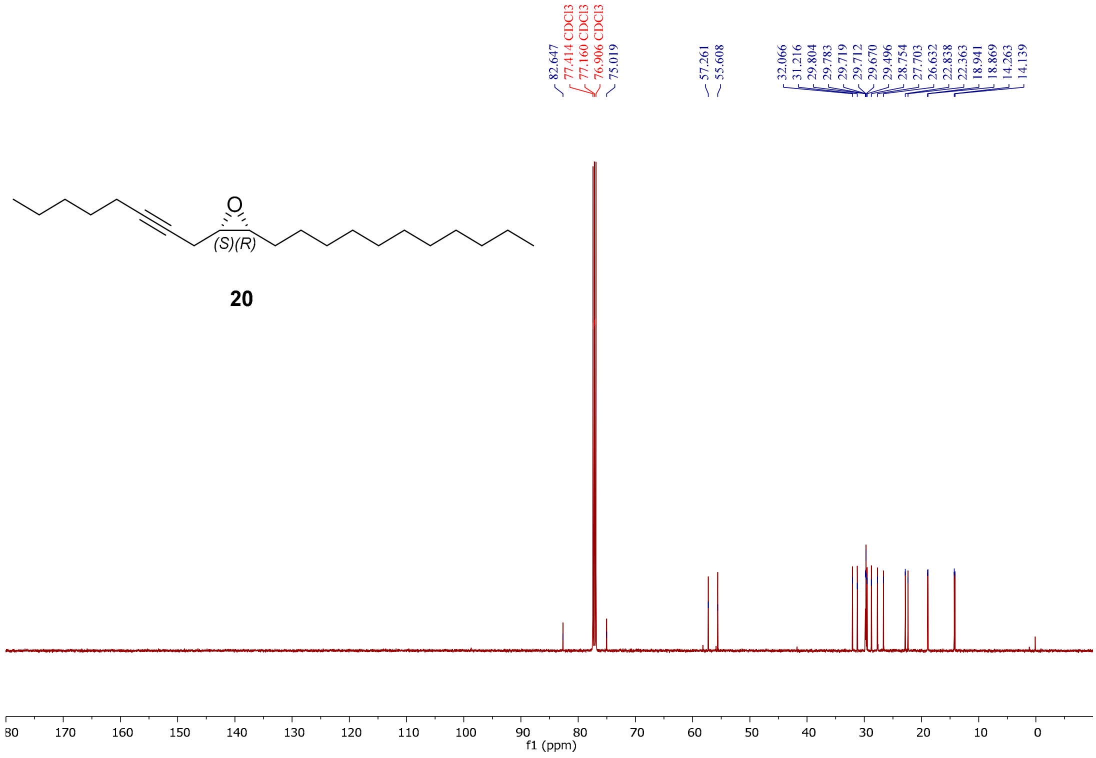

Figure S25. ^{1}H NMR Spectrum of compound **21** (before recrystallization) (500 MHz, $CDCl_3$)

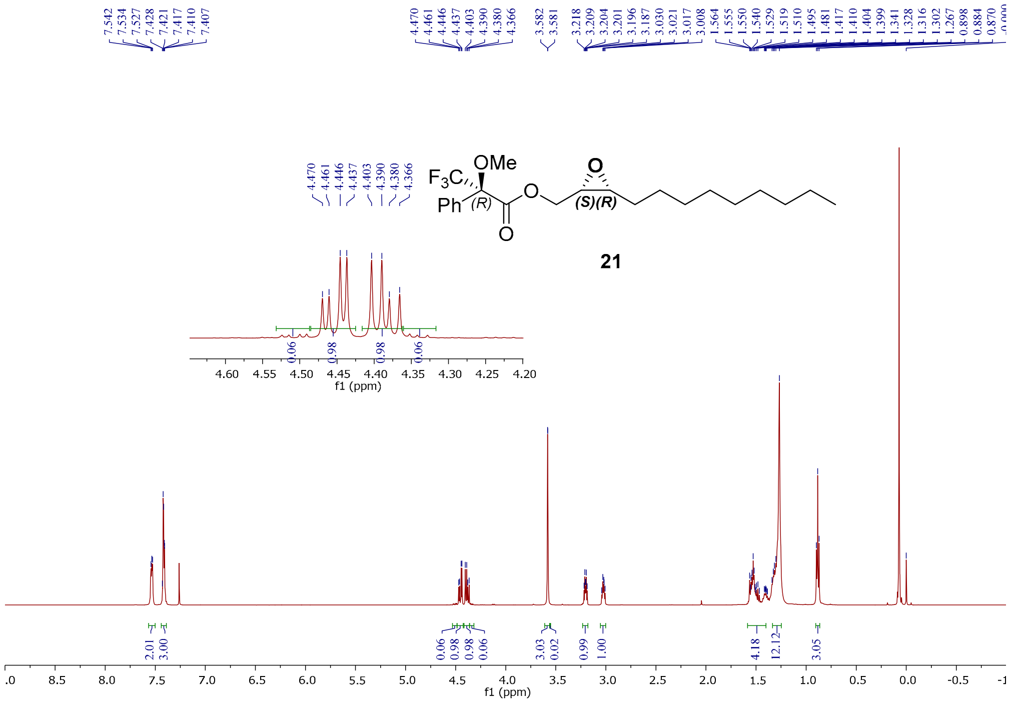

Figure S26. ^{1}H NMR Spectrum of compound **21** (after recrystallization) (500 MHz, $CDCl_3$)

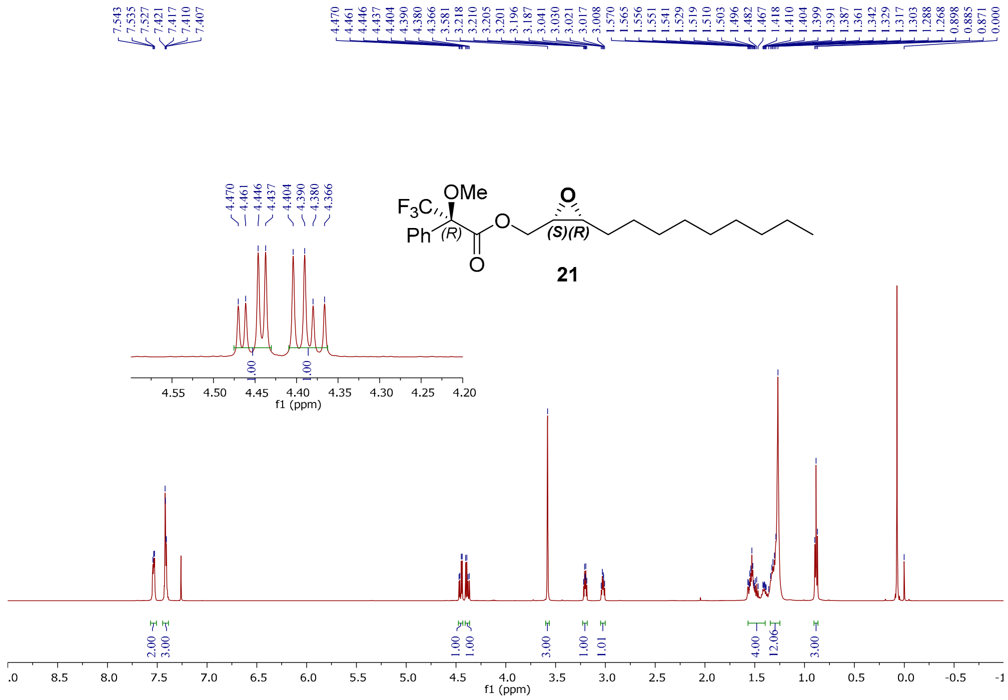

Figure S27. ^{1}H NMR Spectrum of compound **22** (before recrystallization) (500 MHz, $CDCl_3$)

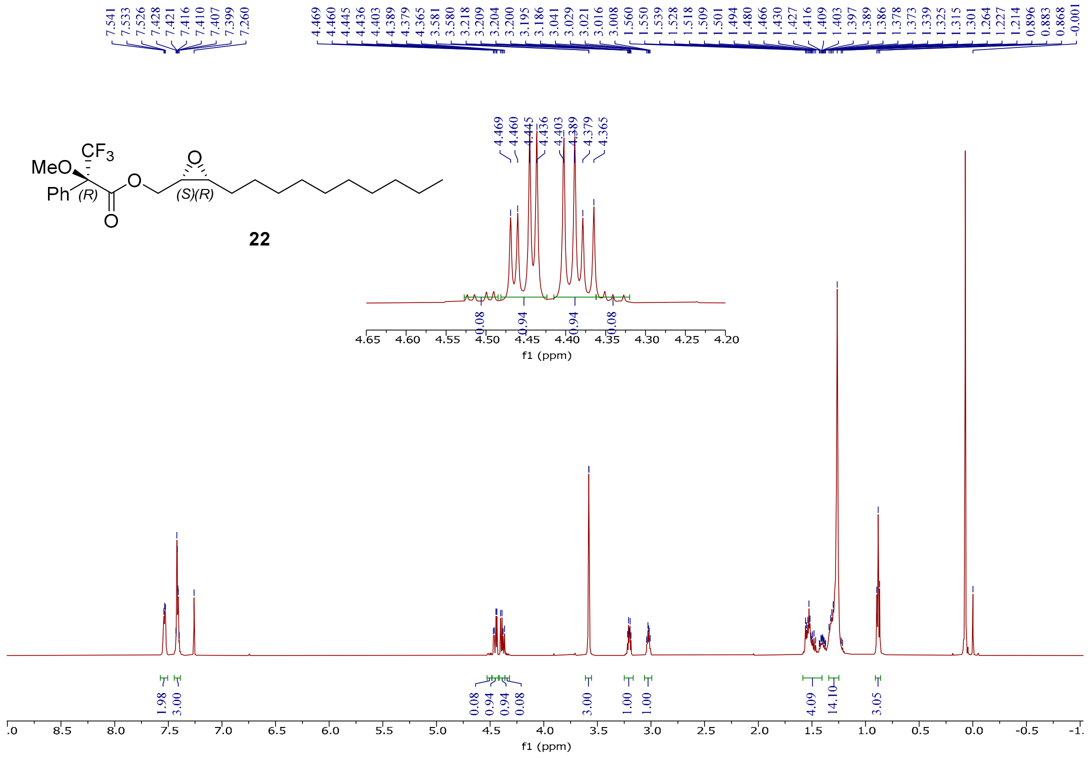

Figure S28. ^{1}H NMR Spectrum of compound **22** (after recrystallization) (500 MHz, $CDCl_3$)

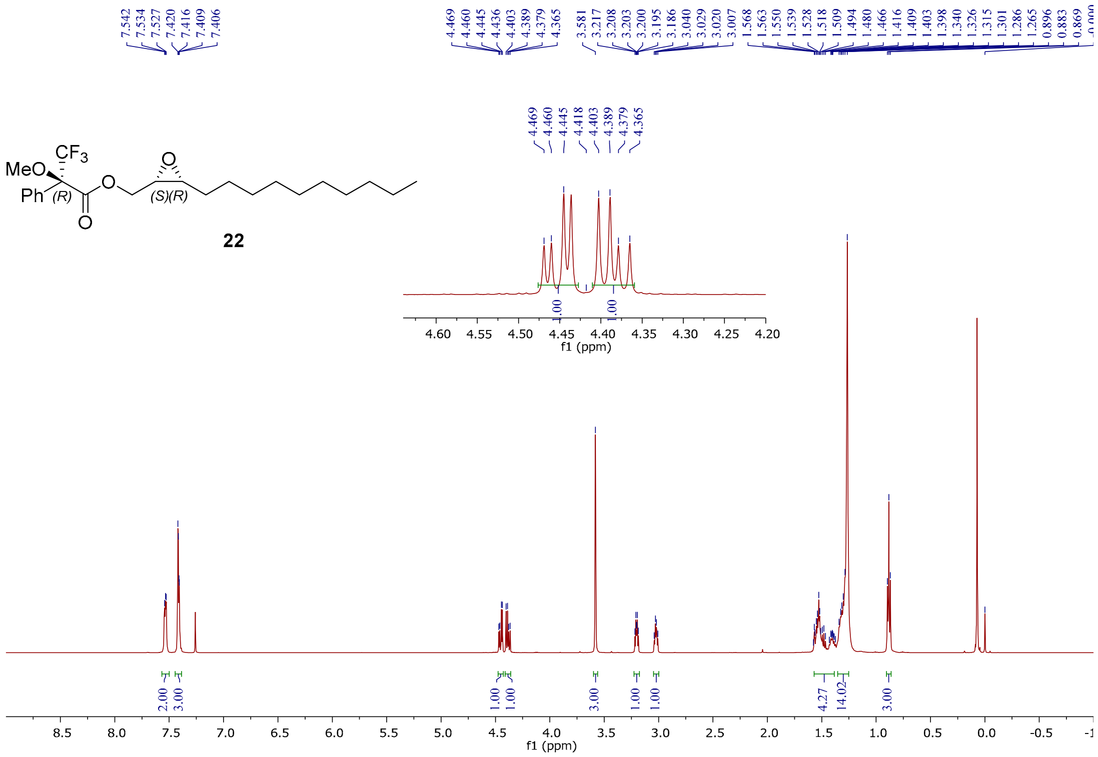

Figure S29. ^{1}H NMR Spectrum of compound **23** (before recrystallization) (500 MHz, $CDCl_3$)

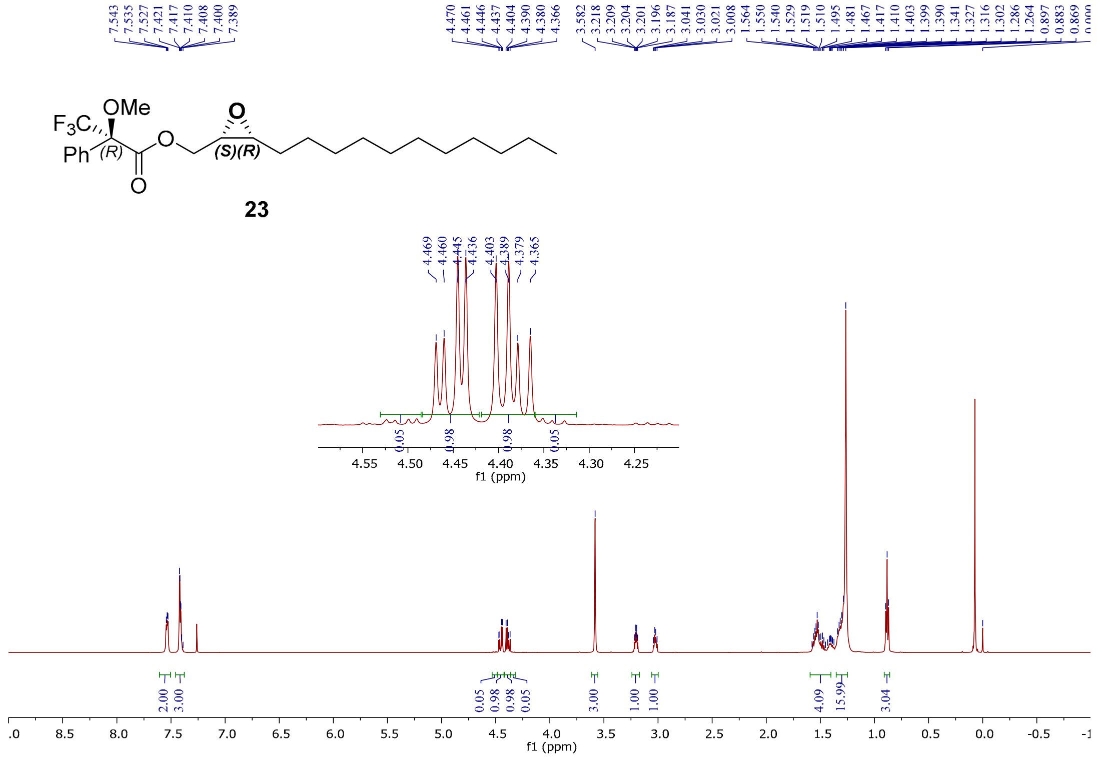

Figure S30. ^{1}H NMR Spectrum of compound **23** (after recrystallization) (500 MHz, $CDCl_3$)

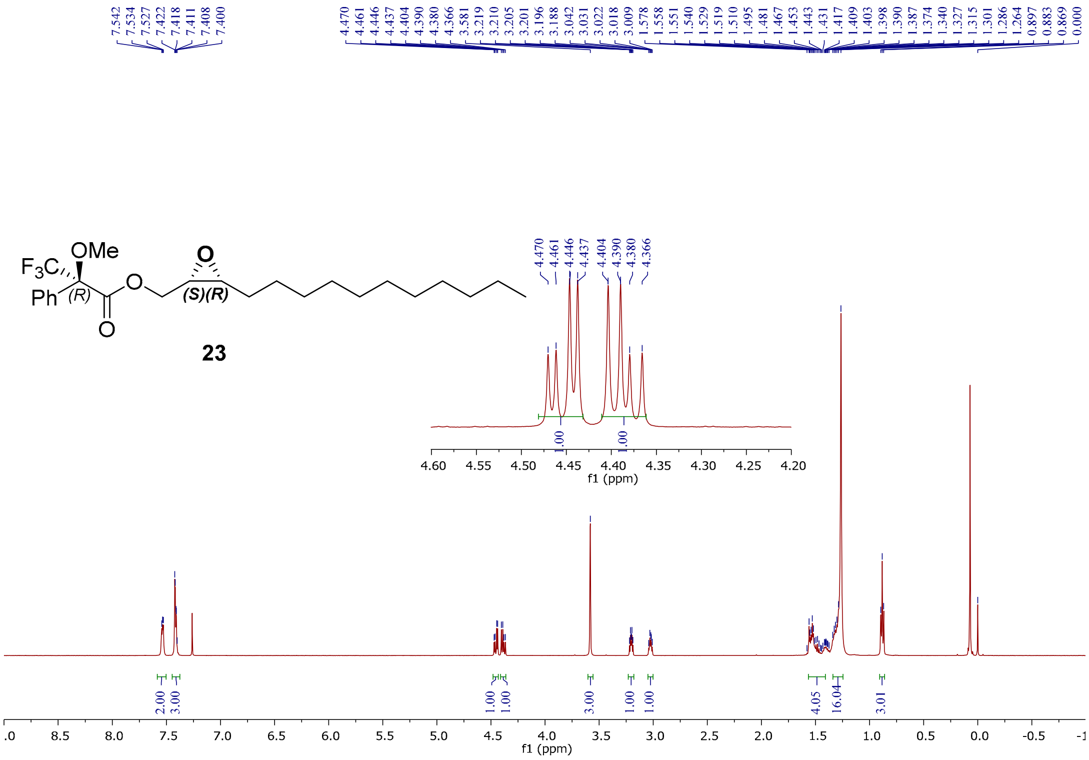

Figure S31. ^{1}H NMR Spectrum of compound **1** (500 MHz, $CDCl_3$)

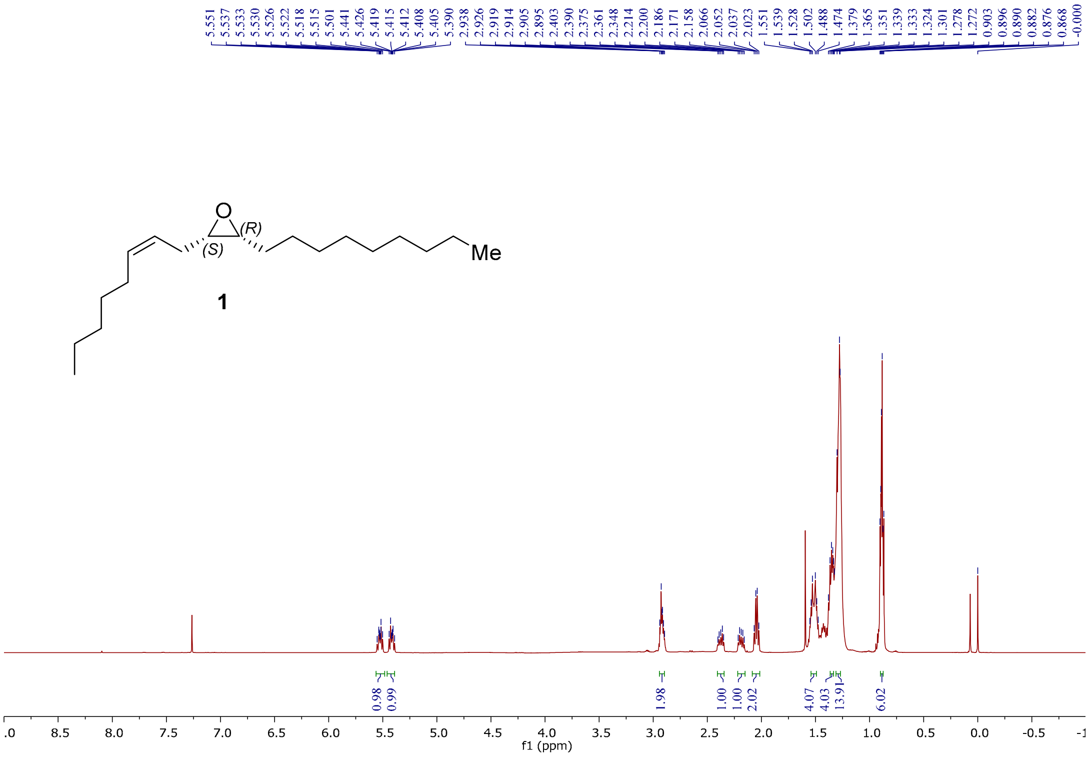

Figure S32 ^{13}C NMR Spectrum of compound **1** (125 MHz, $CDCl_3$)

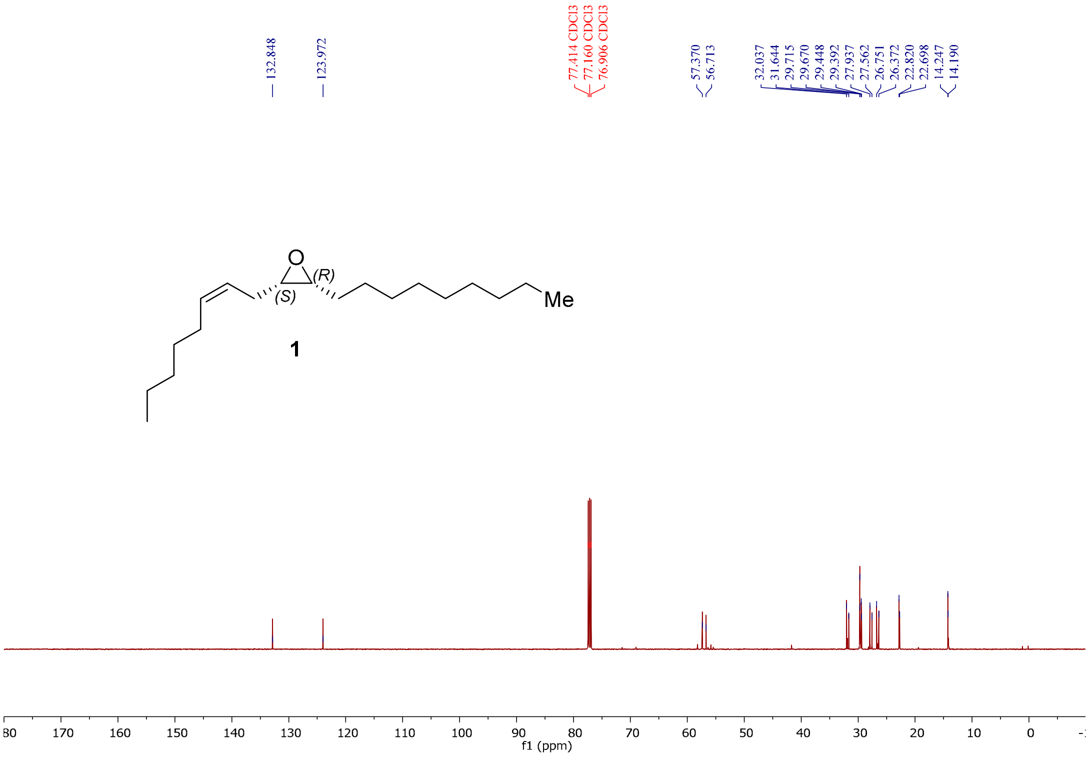

Figure S33. ^{1}H NMR Spectrum of compound **2** (500 MHz, $CDCl_3$)

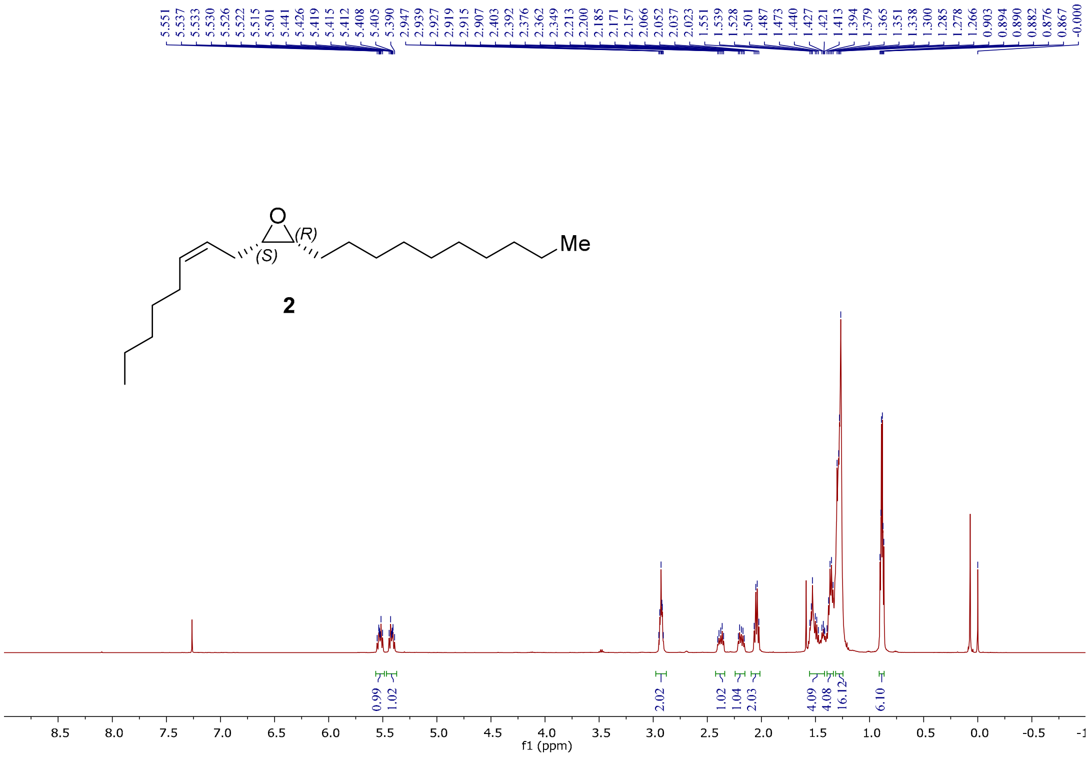

Figure S34 ^{13}C NMR Spectrum of compound **2** (125 MHz, $CDCl_3$)

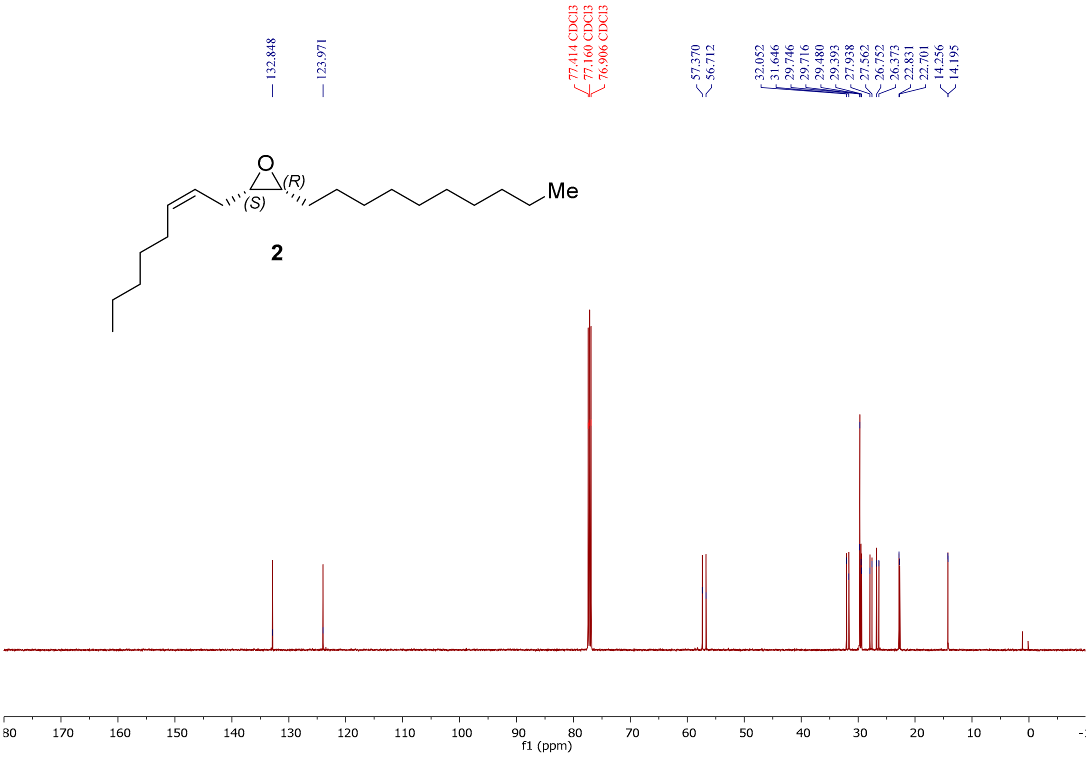

Figure S35. ^{1}H NMR Spectrum of compound **3** (500 MHz, $CDCl_3$)

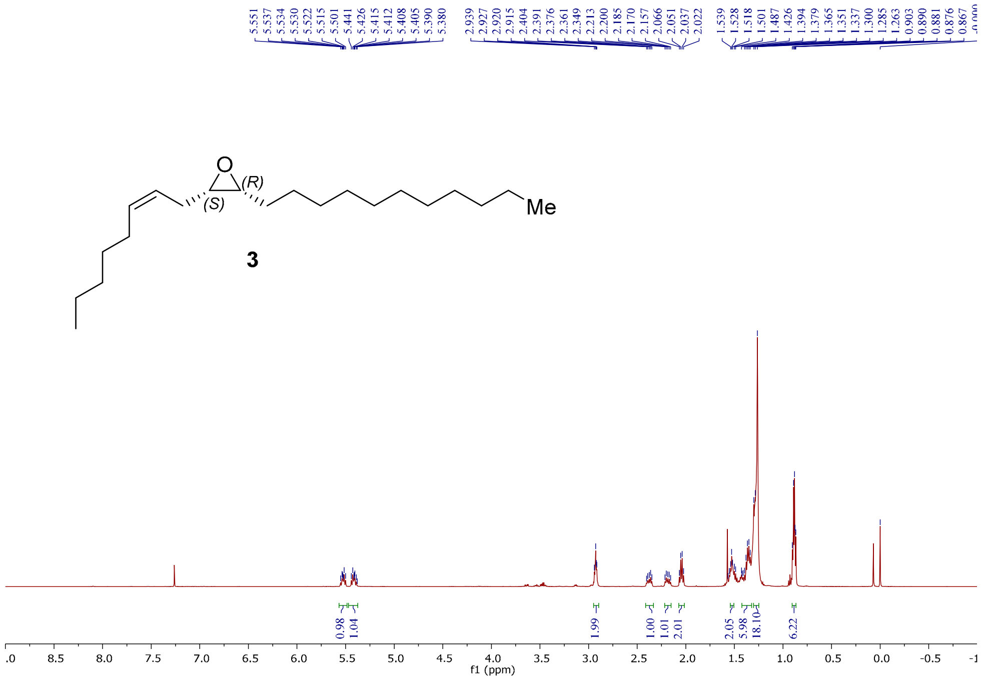

Figure S36 ^{13}C NMR Spectrum of compound **3** (125 MHz, $CDCl_3$)

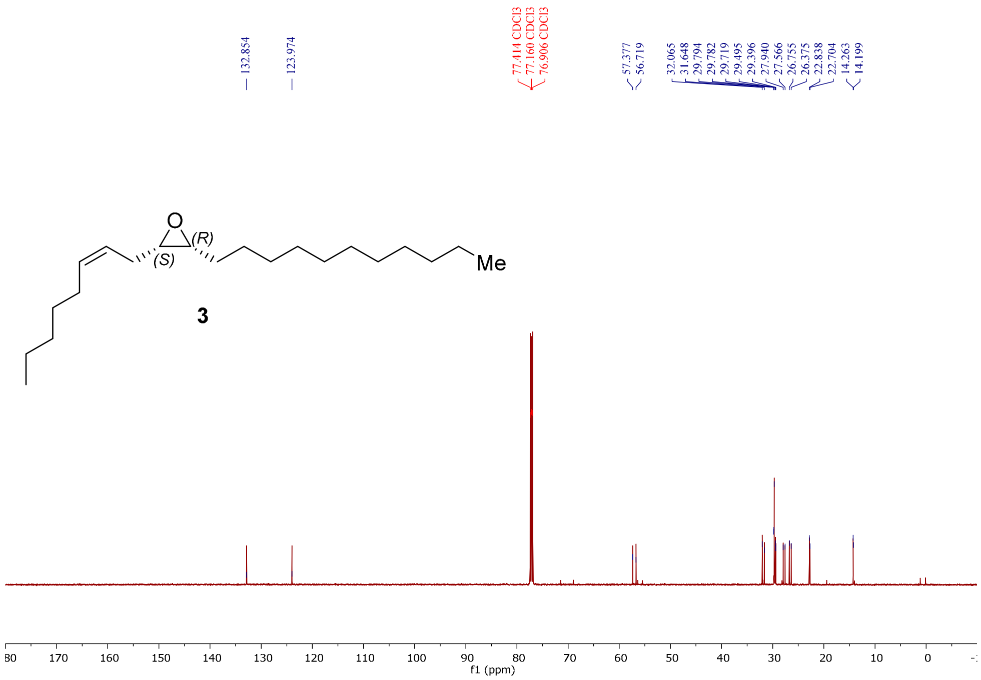

4. Reference

1. Zhou, Y.; Huang, Y.; Li, S.; Yang, P.; Zhong, J.; Yin, J.; Ji, K.; Yang, Y.; Ye, N.; Wang, L. Wang, M.; Wang, M; Bian, Q, Total syntheses of 9-epoxyfalcarindiol and its diastereomer. *Tetrahedron: Asymmetry* . **2017,** *28*, 288-295.
2. Mao, J.; Li, S.; Zhong, J.; Wang, B.; Jin, J.; Gao, Z.; Yang, H.; Bian, Q., Total synthesis of panaxydol and its stereoisomers as potential anticancer agents. *Tetrahedron: Asymmetry* . **2016**, *27*, 69-77.
